# Seroprevalence of toxoplasmosis in pregnant women and its associated factors among hospital and community populations in Lambayeque, Peru

**DOI:** 10.1590/0037-8682-0164-2019

**Published:** 2020-03-16

**Authors:** Heber Silva-Díaz, Emma V. Arriaga-Deza, Virgilio E. Failoc-Rojas, Yessica R. Alarcón-Flores, Sara Y. Rojas-Rojas, Lizzie K. Becerra-Gutiérrez, Katya M. Mera-Villasis, Franklin R. Aguilar-Gamboa, Teresa Silva-García

**Affiliations:** 1 Laboratory of Parasitology, Vector-Borne Diseases and Zoonosis, Hospital Regional Hospital Lambayeque, Lambayeque, Peru.; 2 Unidad de Investigación para la Generación y Sintesis de Evidencias en Salud, Universidad San Ignacio de Loyola, Lima, Peru.; 3 Faculty of Biological Sciences, Universidad Nacional Pedro Ruiz Gallo. Lambayeque, Peru.; 4 Laboratory of Immunology and Virology, Hospital Regional Lambayeque, Lambayeque, Peru.

**Keywords:** Toxoplasmosis, Pregnancy, Seroprevalence, Antibodies

## Abstract

**INTRODUCTION:**

Toxoplasmosis is a zoonosis caused by *Toxoplasma gondii*. This study investigated the prevalence and factors associated with toxoplasmosis among pregnant women.

**METHODS:**

We followed an analytical observational study. From July 2016 to June 2017, 218 pregnant women were selected. The infection was detected through serological dosage of *anti-T.gondii* Immunoglobulin(Ig) M and IgG antibodies.

**RESULTS:**

The seroprevalence was 35.8%; the factors associated with infection were consumption of non-drinking water, residence in an urban area, and threatened abortion during the current pregnancy.

**CONCLUSIONS:**

The seroprevalence of toxoplasmosis among pregnant women is high. The risk factors are dependent on environmental determinants.

Human toxoplasmosis is a re-emerging, cosmopolitan zoonosis caused by *Toxoplasma gondii*. It is an obligate intracellular parasite whose carrier and definite host is the domestic cat and other felines^1^ ^,^ ^2^. It is also transmitted vertically from mother to child^3^ ^,^ ^4^. Primary infection during pregnancy may cause miscarriage, perinatal death, or fetal organ abnormalities, depending on the pregnancy trimester^4^ ^,^ ^5^. One-third of infections in pregnancy may be transmitted to the fetus by the hematogenous spread of *T. gondii* in its invasive form (tachyzoite), which can cross the placental barrier^5^. A fetus exposed to *T. gondii* in the uterus may develop congenital toxoplasmosis with significant visual and neurological consequences^4^.

The actual prevalence of the *Toxoplasma gondii* infection during pregnancy is unknown and varies among countries. Recent studies have estimated the prevalence using the enzyme-linked immunosorbent assay (ELISA). In Iran and Brazil, the frequency of IgG antibodies is 38% and 50%, while that of IgM antibodies is 4% and 90%, respectively^6^ ^,^ ^7^.

The risk factors for toxoplasmosis in pregnancy are debated as some studies consider maternal age >30 years, exposure to cats, and consumption of raw meat and unwashed vegetables as risk factors^8^ ^,^ ^9^. However, these factors were not associated with toxoplasmosis in other studies^6^ ^,^ ^8^.

Nevertheless, in Lambayeque, Peru, the exact prevalence and associated risk factors for toxoplasmosis during pregnancy are unknown. This study aimed to estimate the seroprevalence of toxoplasmosis during pregnancy, and its associated risk factors, among patients admitted to the Regional Hospital of Lambayeque (RHL) and from a community in Lambayeque.

We conducted an observational, cross-sectional study. The population consisted of pregnant women who were treated at the Services of Obstetrics and Gynecology of RHL from July 2016 to June 2017 and pregnant women from the district of Morrope, Lambayeque.

The study was conducted with a sample of 218 pregnant women, estimated from a general population of about 5000 pregnant women (2900 from RHL and 2100 from Morrope), with an error of 0.06, an expected frequency of 30%,^1^ and a 95% confidence interval (95% CI). These women were selected by systematic random sampling, considering the patients’ order of arrival or choosing the first number at random. A 5 mL sample of blood was collected from each patient using a vacuum system. They were administered a questionnaire to collect data related to factors associated with the disease.

Pregnant women from the region of Lambayeque participated in the study voluntarily; they agreed to participate through providing signed consent. Those who were hospitalized or had conditions (mental or physiological) that would prevent the administration of the structured questionnaire or blood collection, were excluded.

The data were collected through random sampling based on the order of arrival to the hospital or nonhospital care services (community). We considered women with positive pregnancy confirmation test results (positive BHCG and first echography). To collect data regarding the associated risk factors, a semi-structured questionnaire was administered to each participant. This instrument was revised and validated by the authors of this study and by three experts (two in gynecology and one in infectology). It was divided into four sections-eight questions about socio-demographic information, eight questions about clinical information, six questions about eating habits and environment, and four questions about the participant’s laboratory values. The last section was completed by the physician and biologist. A pilot study was not conducted due to logistical and funding issues.

The exposure variables studied were: socio-demographic (maternal age, origin, district), clinical aspects (gestational age, parity, threatened abortion defined as heavy menses during the first trimester of the current pregnancy, history of previous abortion defined as the loss of a fetus in a previous pregnancy, preterm babies defined as children born before 37 weeks of gestational age, urinary tract infections, and development of ocular anomalies defined by echography and evidenced by a specialist [gynecologist]), eating habits and behaviors (consumption of drinking water, consumption of raw vegetables, and presence of household dogs and/or cats), and significant outcomes (positive serology to toxoplasmosis). To clarify, in Lambayeque there is access to water; however, some areas do not have access to clean drinking water, or the people are not custom to drinking the clean water.

To determine positive serology, we assessed the presence of serum *anti-T.gondii gondii* antibodies in the sera collected from the patients and stored at -70°C. The serological tests used were standardized using an ELISA, following the manufacturer’s recommendations (Virion-Serion, Germany). We quantitatively evaluated the anti-*T. gondii* IgM and IgG antibodies and the avidity of IgG. A positive interpretation occurred at values higher than 30 IU/mL of IgM and 350 IU/mL of IgG. The sensitivity and specificity of the kits were 97.8% and 95.7% for IgM, and 98.2% and 99.4% for IgG, respectively.

Samples that tested positive for anti-*T. gondii* IgG antibodies qualified for the IgG avidity test; this was used to determine the time of seroconversion, suggesting that there was a recent infection if avidity was lower than 45%. Seropositivity for toxoplasmosis was presumed when the tests were positive for one or more of the markers (IgM or IgG). Patients with a positive result were referred to infectology and gynecology for proper management of the infection.

Data were analyzed using the statistical software, Stata^®^ 14.0 (StatCorp, Texas, US), according to the characteristics of each variable. We conducted a descriptive analysis of qualitative variables using absolute and relative frequencies with 95% CIs. The quantitative variables were presented as measures of central tendency and dispersion, producing tables for analysis. Normality was previously assessed using the Shapiro-Wilk test.

The bivariate analysis of the association between toxoplasmosis and the risk factors was conducted using the Pearson’s Chi-square test or the Fisher’s exact test, with a significance level of <0.05. Measures of association between the risk factors and the seroprevalence of toxoplasmosis were presented as prevalence ratios (PR) with 95% confidential interval (CI). A logistic regression was used with a generalized linear model (Poisson family), adjusted by the cluster of the origins of the pregnant women; the variables were analyzed using a likelihood ratio test lower than 0.20 in the raw analysis.

We were granted a permit from the RHL to conduct this research and approval from the Ethics Committee by certificate of approval: 03222-86-14 CEI from the RHL. Throughout the study, we ensured that autonomy and anonymity were maintained through providing informed consent to each participant.

We studied 218 pregnant women: 115 (52.8%) were recruited from hospitals and 103 (47.3%) were recruited from the community. The overall median age was 25.5 years old (RIQ: 10) and the median number of pregnancies was 2 (RIQ: 2). Of these women, 78 (35.8%; 95% CI: 29.4% to 42.1%) tested positive for IgG or IgM. Of these, 66 (30.3%) had *anti-T.gondii* high-avidity IgG antibodies and 12 (5.5%) had *anti-T.gondii* IgM and/or *anti-T.gondii* low-avidity IgG antibodies. Only 5 cases (2.3%) had low-avidity IgG antibodies.

The median age of the pregnant women who were seropositive for toxoplasmosis was 27 years old; this was greater than the overall median age of 25.5 years. The positive seroprevalence was 39.8% among the hospital population and 32.2% among the community population; however, there was no difference between them (p=0.259; Table 1).

**TABLE 1:** Clinical-epidemiological characteristics of pregnant women treated in a hospital or in the community of Lambayeque, Peru between 2016 and 2017

	Community		Hospital		Total	
Characteristic	n	%	n	%	n	%
Age (years)*	28	(9)	24.7	(11)	25.5	(10)
**Area or residence**						
Urban	96	100	0	0	96	44.1
Rural	7	5.74	115	94.26	122	55.9
**Type of water used**						
Non-drinking	60	44.12	76	55.88	136	62.4
Drinking	43	52.44	39	47.56	82	37.69
**Having household cats**						
Yes	27	36.99	46	63.01	73	33.5
No	76	52.41	69	47.59	145	66.5
**Having household dogs**						
Yes	45	39.47	69	60.53	114	52.3
No	58	55.77	46	44.23	104	47.7
**Consumption of raw vegetables**						
Yes	88	44.9	108	55.1	196	89.9
No	15	68.18	7	31.82	22	10.1
**Gestational age**						
First trimester	17	29.31	41	70.69	58	26.6
Second trimester	32	47.76	35	52.24	67	30.8
Third trimester	54	58.06	39	41.94	93	42.6
**Threatened abortion**						
Yes	16	59.26	11	40.74	27	12.38
No	53	33.76	104	66.24	157	72.02
Not mentioned	31	100	0	0	34	15.6
**Threatened abortion**						
Yes	35	70	15	30	50	22.9
No	68	40.48	100	59.52	168	77.1
**Preterm babies**						
Yes	3	14.29	18	15.65	21	9.63
No	65	40.12	97	59.88	162	74.31
Not mentioned	35	35	0	0	35	16.05
**Urinary tract infection**						
Yes	14	66.67	7	33.33	21	9.63
No	125	63.45	72	36.54	191	90.4
**Newborn with ocular anomalies**						
Yes	3	11.11	24	88.89	27	12.4
No	80	41.88	111	58.12	191	87.6

*Non-parametric variable, Shapiro-Wilk <0.05. Age is reported by median and the interquartile range.

The bivariate analysis showed that consumption of non-drinking water was associated with the frequency of toxoplasmosis (p=0.015; Prevalence Ratio (PR)=1.63; 95% CI: 1.07-2.48). (Table 2). 

**TABLE 2:** Frequency and bivariate analysis of the presence of *anti-T.gondii* antibodies among pregnant women treated in a hospital or in the community of Lambayeque, Peru from 2016 to 2017.

	Positive to toxoplasma^+^ (%)		Negative to toxoplasma (%)		Crude model	p-value
Characteristic	n	%	n	%	PR [95% CI]	
**Total**	78 (35.8%)		140 (64.2%)			
Age (years)*	27 [23-33]		27 [23-33]		1.02 [0.97 - 1.08]	0.328
**Area of residence** ^‡^						
Urban	38 (39.6)		58 (60.4)		1.2 [0.84 - 1.72]	0.298
Rural	40 (32.8)		82 (67.2)		1	
**Type of water used** ^‡^						
Non-drinking	57 (41.9)		79 (58.1)		1.63 [1.07 -2.48]	0.015
Drinking	21 (25.6)		61 (74.4)		1	
**Having household cats** ^‡^						
Yes	24 (32.9)		49 (67.1)		0.88 [0.59 -1.30]	0.525
No	54 (37.2)		91 (62.8)		1	
**Having household dogs** ^‡^						
Yes	43 (37.7)		71 (62.3)		1.12 [0.78-1.6]	0.531
No	35 (33.7)		69 (66.3)		1	
**Consumption of raw vegetables** ^‡^						
Yes	72 (36.7)		124 (63.3)		1.34 [0.66-2.73]	0.38
No	6 (27.3)		16 (72.7)		1	
**Gestational age** ^‡^						
First trimester	20 (35.1)		37 (64.9)		1	
Second trimester	26 (38.2)		41 (61.8)		1.06 [0.57 - 1.84]	0.919
Third trimester	32 (34.4)		61 (65.6)		0.95 [0.54 - 1.64]	0.856
**Threatened abortion** ^‡^						
Yes	13	17.11	63	82.89	1.43 [0.92 - 2.23]	0.136
No	14	10.7	125	89.93	1	
**History of abortion** ^‡^						
Yes	15	30	35	70	0.8 [0.51 - 1.27]	0.331
No	63	37.5	105	62.5	1	
**Preterm babies** ^‡^						
Yes	8	38.1	13	61.9	1.03 [0.45 -2.31]	0.948
No	70	35.5	92	64.5	1	
**Urinary tract infection** ^‡^						
Yes	7	33.3	14	66.7	0.94 [0.49 - 1.79]	0.867
No	71	36	126	64	1	
**Newborn with ocular anomalies** ^‡^						
Yes	11	40.7	16	59.3	1.23 [0.74 -2.04]	0.441
No	67	35.1	124	64.9	1	

*The median is used because it is a non-parametric variable (Kolmorov-Smirnov test<0.05). **‡**The Chi-square test was used. ^+^Positive meant there were anti-*T. gondii* IgM or IgG antibodies. ***Anti-T. gondii*:** *Anti-Toxoplasma gondii*; **PR:** Prevalence ratio; **CI:** Confidence interval.

The most relevant clinical aspects of the pregnant women were history of threatened abortion (n=50; 22.9%) and threatened abortion in the current pregnancy (n=27; 14.6%). Having a threatened abortion was associated with a higher frequency of serology compatible with a recent infection (p<0.01; PRa=1.38, 95% CI: 1.12-1.71; Table 3) compared to have no threatened abortion.

In the multivariate analysis, we observed a 20% higher risk for seroprevalence of toxoplasmosis when living in an urban area compared to living in a rural area (95% CI: 1.15-1.26). 

There was an 80% higher risk of having seroprevalence of toxoplasmosis when consuming non-drinking water, and a 36% higher risk when abortion was threatened (Table 3). 

**TABLE 3:** Factors associated with the presence of *anti-T.gondii* antibodies among pregnant women treated in a hospital and in the community of Lambayeque, Peru from 2016 to 2017.

	Adjusted model*	P-value
	RPa [95% CI]	
**Area of residence**		
Urban	1.20 [1.13 - 1.30]	0.001
Rural	ref.	
**Type of water used**		
Non-drinking	1.80 [1.67 -1.94]	0.001
Drinking	Ref.	
**Having household cats**		
Yes	0.94 [0.82 -1.07]	0.354
No	Ref.	
**Threatened abortion**		
Yes	1.38 [1.12 - 1.71]	0.003
No	Ref.	

*We conducted a generalized linear model with Poisson family, adjusted by the variables presented, and adjusted by cluster. **+**We only included variables with a likelihood ratio test lower than 0.20 in the raw analysis (consumption of non-drinking water, threatened abortion), and variables that may be explained by mechanisms of pathogenicity (area of residence, household cats). *Anti-T. gondii*: *Anti-Toxoplasma gondii*. **PRa:** Prevalence ratio adjusted; **CI:** Confidence interval; **Ref:** referrer.

The prevalence of *anti-T.gondii* antibodies in this study was 35.8%. The seroprevalence is similar to values found in other regions-30% in Colombia^1^, 38% in Iran^6^, 43.5% in Aracaju, Brazil^10^, 31% in Sri Lanka^11^, and 30% in Burkina Faso^12^. However, in regions where toxoplasmosis is endemic, seropositivity for toxoplasmosis in pregnant women was even higher (50% in Sao Paulo, Brazil^7^ and 89% in Goiania, Brazil^13^). In our study, the prevalence of patients whose serology was compatible with an acute infection was 5.5%; this is similar to other studies^1^ ^,^ ^6^ ^,^ ^7^. 

Understanding the socio-demographic profile of the population of child-bearing age women is essential for formulating primary and secondary health-care actions to treat toxoplasmosis. This protozoan can be vertically transmitted to the developing fetus, causing a serious, debilitating disease^14^. The risk for congenital toxoplasmosis (e.g., leading to chorioretinitis, brain calcification, hydrocephalus, microcephaly, or macrocephaly) is proportional to the gestational age at the time of seroconversion. The risk for fetal compromise (e.g., abortion or death), however, has an inverse relationship with gestational age^3^ ^,^ ^5^ ^,^ ^11^.

The current study reported a higher seroprevalence among participants from urban areas than among those from rural areas. This may be due to urbanization in developing countries, which has been associated with poor socio-economic conditions caused by overpopulation and poverty and factors associated with a higher seroprevalence (e.g., poor health, inadequate control of food sources, etc.). This suggests that factors affecting the risk of infection are more complex than a simple urban-rural division and may be confounded by other factors. This association was also found in other studies from Iran and Brazil^6^ ^,^ ^11^ ^-^ ^13^.

Through multivariate analysis, we found that the presence of cats had no association with the seroprevalence of toxoplasmosis in pregnancy. No association was also observed in other studies investigating the association between seroprevalence and the presence of household cats in Brazil^10^ ^,^ ^13^. However, some studies from Burkina Faso and Iran have shown an association^6^ ^,^ ^12^. The association between the presence of household cats and toxoplasmosis is difficult to assess using epidemiological studies as it depends on direct exposure to cat feces (in this study we only considered the presence of household cats). An explanation for the lack of association between the presence of household cats and seropositivity may be that people with household cats may be more careful to eliminate and avoid exposure to cat feces.

Threatened abortion during pregnancy was a risk factor. In previous studies, threatened abortion during the current pregnancy had not been assessed, but history of previous miscarriage was^6^ ^,^ ^13^. It is important to consider this factor and further investigate the possible associations between a threatened abortion during the current pregnancy and seropositivity for toxoplasmosis. 

About diagnoses, this study provided no further clinical usefulness (approximately 90% of cases go unnoticed). However, improving the epidemiological understanding of the disease can be useful to indicate that there is a highly prevalent parasite among the population that has a great risk to those who are pregnant. Laboratory diagnosis is still important^5^ ^,^ ^6^.

We acknowledge that there are some limitations in our study. First, the evaluation of the risk factors was conducted based on responses from the study participants; there could be measurement bias. Second, the study sample was from hospital and community populations; hence, the prevalence cannot be generalizable to the entire population. The prevalence of seropositivity for toxoplasmosis in pregnant women is approximated. 

We suggest that preventive measures should be continued (e.g., educating pregnant women about toxoplasmosis and its transmission routes and preventative methods). Greater efforts should be made to improve prevention and surveillance for this group of patients as congenital toxoplasmosis is still a neglected disease in Peru, despite the current efforts^15^.

In conclusion, positive seroprevalence of *T. gondii* is common among pregnant women in Lambayeque. We found that older maternal age, urban origin, consumption of non-drinking water, and threatened abortion during the current pregnancy are risk factors associated with toxoplasmosis. We recommend that these factors be monitored among women of child-bearing age (the population most at risk). 
